# Supplementary material for: Relationship between the Blood Urea Nitrogen to Creatinine Ratio and In-Hospital Mortality in Non-Traumatic Subarachnoid Hemorrhage Patients: Based on Propensity Score Matching Method
Source: J Clin Med. 2022 Nov 28;11(23):7031. doi: 10.3390/jcm11237031 (PMC9736588; doi:10.3390/jcm11237031)
Supplement: Supplementary file 1 [file jcm-11-07031-s001.zip › jcm-1982130-supplementary-Table S1.pdf]

Table S1: The best cut-off value, specificity, sensitivity and Youden Index of UCR.

Cut-off value	Sensitivity	1 - Specificity	Youden Index
27.20779200	0.217	0.071	0.146
26.54761900	0.222	0.084	0.138
26.90476200	0.217	0.079	0.138
26.39610350	0.222	0.085	0.137
26.30681800	0.222	0.087	0.135
26.12500000	0.222	0.089	0.133
24.33035700	0.259	0.130	0.130
25.66964300	0.228	0.100	0.128
25.85714300	0.222	0.095	0.128
24.72222200	0.254	0.127	0.127
25.59027800	0.228	0.102	0.125
24.40972200	0.254	0.130	0.124
25.11904750	0.233	0.109	0.124
24.08333350	0.270	0.146	0.123
25.44444450	0.228	0.108	0.120
24.22619050	0.265	0.145	0.119
27.33201550	0.190	0.071	0.119
25.28571400	0.228	0.109	0.119
21.48351650	0.360	0.244	0.116
21.60256450	0.354	0.244	0.111
21.74242450	0.344	0.233	0.111
21.90909100	0.339	0.228	0.111
27.75000000	0.180	0.070	0.110
23.69318200	0.286	0.176	0.110
27.44565200	0.180	0.071	0.109
28.16666650	0.175	0.066	0.109
23.60389650	0.286	0.177	0.108
23.45238100	0.286	0.179	0.107
23.87500000	0.280	0.175	0.106
21.33928550	0.360	0.256	0.103
17.63888900	0.534	0.433	0.102
21.05555550	0.365	0.264	0.101
17.20779200	0.561	0.461	0.100
21.18055550	0.360	0.260	0.099
17.88888900	0.524	0.425	0.099
16.79487200	0.582	0.483	0.099
22.36111100	0.317	0.219	0.099
17.30303000	0.556	0.457	0.098
20.91666650	0.365	0.267	0.098
28.66071450	0.159	0.061	0.098
22.11111100	0.317	0.220	0.097

20.77380950	0.365	0.268	0.097
22.79220800	0.312	0.216	0.096
20.35714300	0.365	0.269	0.096
28.45238100	0.159	0.063	0.095
18.66071450	0.471	0.376	0.095
18.78676450	0.460	0.365	0.095
22.61363650	0.312	0.218	0.095
16.30681800	0.614	0.519	0.094
16.39610350	0.608	0.514	0.094
17.41666650	0.550	0.457	0.093
19.04545450	0.450	0.358	0.092
15.10000000	0.693	0.601	0.092
18.45238100	0.476	0.385	0.091
18.94444450	0.450	0.359	0.091
17.07142850	0.571	0.481	0.091
19.18060200	0.439	0.348	0.091
23.20512800	0.291	0.201	0.090
18.85620900	0.455	0.365	0.090
19.11067200	0.439	0.350	0.089
23.03846150	0.291	0.202	0.089
16.54761900	0.603	0.514	0.089
28.87500000	0.148	0.060	0.089
16.96153850	0.571	0.483	0.088
18.09090900	0.497	0.409	0.088
18.28431350	0.492	0.404	0.088
15.26666650	0.688	0.601	0.087
16.13247850	0.624	0.538	0.087
19.36538450	0.434	0.347	0.087
18.20855600	0.492	0.405	0.087
22.91979950	0.296	0.210	0.086
16.05555550	0.624	0.539	0.085
15.35897400	0.683	0.601	0.082
19.51190500	0.429	0.347	0.081
22.99122800	0.291	0.210	0.081
15.77380950	0.651	0.570	0.081
29.50000000	0.138	0.060	0.078
15.43560600	0.677	0.600	0.078
15.50505050	0.672	0.595	0.077
15.40064100	0.677	0.601	0.076
16.20192300	0.614	0.538	0.076
19.76190500	0.423	0.347	0.076
15.85784300	0.640	0.569	0.072
30.16129050	0.116	0.045	0.071
31.16883100	0.111	0.041	0.070

15.63492100	0.656	0.587	0.069
30.83916100	0.111	0.043	0.068
14.49494950	0.720	0.653	0.067
15.94117650	0.635	0.569	0.066
30.54590600	0.111	0.045	0.066
33.55731250	0.090	0.026	0.064
14.85294100	0.714	0.652	0.063
14.36507900	0.730	0.668	0.062
31.54761900	0.101	0.039	0.062
31.83333350	0.095	0.034	0.062
33.40579700	0.090	0.028	0.061
14.62566850	0.714	0.653	0.061
34.14285700	0.085	0.023	0.061
33.87500000	0.085	0.025	0.060
32.66666650	0.090	0.031	0.059
33.69318200	0.085	0.026	0.059
34.72222200	0.079	0.021	0.059
34.36507900	0.079	0.022	0.057
37.32142850	0.058	0.012	0.047
35.31250000	0.063	0.018	0.045
36.90476200	0.058	0.014	0.044
36.19047650	0.058	0.017	0.041
35.66964300	0.058	0.018	0.040
14.08333350	0.735	0.696	0.040
37.75000000	0.048	0.012	0.036
14.22619050	0.730	0.696	0.035
13.92307700	0.741	0.709	0.032
39.00000000	0.042	0.010	0.032
13.79807700	0.741	0.710	0.031
40.83333350	0.037	0.008	0.029
13.69318200	0.751	0.724	0.027
12.88018450	0.799	0.775	0.024
42.08333350	0.032	0.008	0.024
12.61363650	0.815	0.793	0.022
13.60389650	0.751	0.729	0.022
12.79220800	0.810	0.789	0.021
12.93309450	0.794	0.773	0.020
43.92857150	0.026	0.006	0.020
13.55042050	0.751	0.732	0.019
42.67857150	0.026	0.008	0.019
12.11111100	0.836	0.817	0.019
13.43137250	0.751	0.733	0.018
11.44957950	0.868	0.850	0.018
12.40384600	0.825	0.808	0.017

12.26495700	0.825	0.810	0.016
12.98148150	0.788	0.773	0.015
45.35714300	0.021	0.006	0.015
11.62280700	0.862	0.848	0.014
13.02777800	0.778	0.764	0.014
11.52476750	0.862	0.850	0.013
46.57142850	0.016	0.004	0.012
45.85714300	0.016	0.005	0.011
13.19444450	0.772	0.764	0.008
53.33333350	0.011	0.003	0.008
49.40476200	0.011	0.004	0.007
71.66666650	0.005	0.000	0.005
11.74242450	0.841	0.837	0.004
59.66666650	0.005	0.001	0.004
55.50000000	0.005	0.003	0.003
10.15625000	0.905	0.904	0.001
11.90909100	0.836	0.835	0.000
81.00000000	0.000	0.000	0.000
4.95945950	0.995	0.999	-0.004
10.46875000	0.899	0.904	-0.005
10.89285700	0.894	0.899	-0.005
4.62162150	0.995	1.000	-0.005
8.48571450	0.963	0.969	-0.006
8.66071450	0.958	0.964	-0.006
8.36666650	0.963	0.970	-0.007
10.77380950	0.894	0.902	-0.007
9.14545450	0.947	0.955	-0.008
11.18055550	0.878	0.886	-0.008
10.69047650	0.894	0.903	-0.009
11.33928550	0.868	0.877	-0.009
10.64583350	0.894	0.904	-0.010
10.97619050	0.889	0.899	-0.010
9.04545450	0.947	0.959	-0.011
8.94444450	0.947	0.960	-0.013
11.05555550	0.884	0.896	-0.013
9.35609750	0.942	0.955	-0.013
8.25757550	0.963	0.978	-0.015
8.81944450	0.947	0.962	-0.015
8.09090900	0.963	0.979	-0.016
6.37500000	0.979	0.996	-0.017
9.75609750	0.937	0.955	-0.018
6.20192300	0.979	0.997	-0.019
5.57692300	0.979	0.999	-0.020
6.85185200	0.974	0.995	-0.021

7.32142850	0.968	0.990	-0.021
6.58333350	0.974	0.996	-0.023
7.75000000	0.963	0.987	-0.024
7.08994700	0.968	0.995	-0.027
