# Supplementary material for: Relationship between the Blood Urea Nitrogen to Creatinine Ratio and In-Hospital Mortality in Non-Traumatic Subarachnoid Hemorrhage Patients: Based on Propensity Score Matching Method
Source: J Clin Med. 2022 Nov 28;11(23):7031. doi: 10.3390/jcm11237031 (PMC9736588; doi:10.3390/jcm11237031)
Supplement: Supplementary file 1 [file jcm-11-07031-s001.zip › jcm-1982130-supplementary-Table S2.pdf]

Table S2| Univariate logistic regression analyses for in-hospital mortality in patients with non-traumatic subarachnoid hemorrhage.

Variable	OR 95 CI%	P value
Age	1.03 (1.02~1.05)	<0.001
Gender	0.86 (0.62~1.18)	0.343
Ethnicity		
White	0.29 (0.14~0.59)	0.001
Other	0.67 (0.33~1.38)	0.28
Myocardial infarct	1.86 (1.12~3.09)	0.017
Congestive heart failure	1.94 (1.2~3.15)	0.007
Peripheral vascular disease	0.7 (0.39~1.27)	0.241
Dementia	0.87 (0.25~3.07)	0.833
Chronic pulmonary disease	1.49 (0.98~2.27)	0.059
Peptic ulcer disease	0.37 (0.05~2.87)	0.34
Paraplegia	1.07 (0.7~1.63)	0.772
Renal disease	3.29 (2.03~5.35)	<0.001
Malignant cancer	1.77 (0.86~3.66)	0.121
Mild liver disease	3.85 (2.07~7.18)	<0.001
Diabetes	1.44 (0.97~2.15)	0.071
DCI	0.39 (0.17~0.91)	0.03
Sepsis	2.73 (1.94~3.85)	<0.001
Vasospasm	0.25 (0.1~0.63)	0.003
HR	1.02 (1.01~1.04)	<0.001
SBP	1 (0.99~1.01)	0.688
DBP	0.99 (0.97~1)	0.117
MBP	0.99 (0.97~1.01)	0.396
RR	1.18 (1.12~1.23)	<0.001
Temperature	0.88 (0.66~1.18)	0.398
Spo2	1.06 (0.97~1.15)	0.197
Glucose	1 (1~1)	0.723
Hemoglobin	0.93 (0.87~1.01)	0.082
Platelets	1 (1~1)	0.003
WBC	1.07 (1.05~1.1)	<0.001
Anion-gap	1.15 (1.1~1.2)	<0.001
Bicarbonate	0.95 (0.9~1)	0.032
Calcium	1.15 (0.92~1.46)	0.224
Chloride	1.09 (1.06~1.12)	<0.001
Sodium	1.15 (1.11~1.19)	<0.001
INR	1.29 (1.08~1.53)	0.005
PT	1.02 (1.01~1.04)	0.006
APTT	1.01 (1~1.01)	0.008
ALT	1 (1~1)	0.072
AST	1 (1~1)	0.089
OASIS	1.13 (1.11~1.16)	<0.001
GCS	0.84 (0.81~0.88)	<0.001

SOFA	1.33 (1.19~1.49)	<0.001
WFNS		
II	0.04 (0.02~0.1)	<0.001
III	0 (0~Inf)	0.98
IV	0.27 (0.16~0.46)	<0.001
V	1.22 (0.8~1.85)	0.349
Length of ICU stay	0.93 (0.91~0.95)	<0.001
Length of hospital stay	0.95 (0.93~0.98)	<0.001

Abbreviations: HR, heart rate; SBP, systolic blood pressure; DBP, diastolic blood pressure; MBP, mean blood pressure; RR, respiratory rate; SpO2, percutaneous oxygen saturation; DCI, delayed cerebral ischemia; UCR, BUN/creatinine; INR, international normalized ratio; PT, prothrombin time; APTT, activated partial thromboplastin time; ALT, alanine aminotransferase; AST, aspartate aminotransferase; SOFA, sequential organ failure assessment score; GCS, Glasgow coma score; OASIS, oxford acute severity of illness score; ICU, Intensive care unit; WFNS, World Federation of Neurosurgical Societies.
