# Supplementary figures and images for: Relationship between the Blood Urea Nitrogen to Creatinine Ratio and In-Hospital Mortality in Non-Traumatic Subarachnoid Hemorrhage Patients: Based on Propensity Score Matching Method
Source: J Clin Med. 2022 Nov 28;11(23):7031. doi: 10.3390/jcm11237031 (PMC9736588; doi:10.3390/jcm11237031)

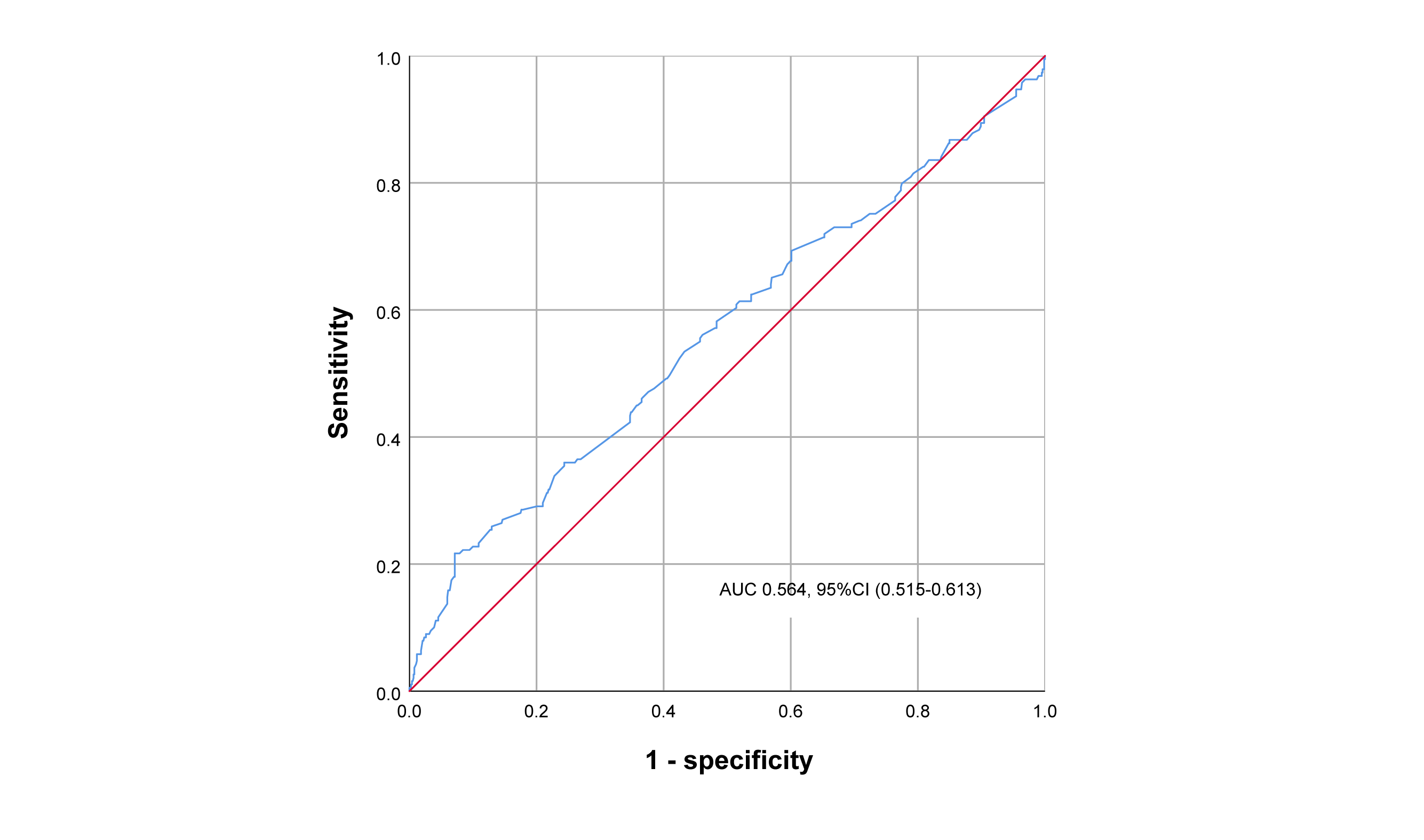

Supplement: Supplementary file 1 [file jcm-11-07031-s001.zip › jcm-1982130-supplementary-Figure S1. The ROC curve of the UCR.tif]
